# Population Pharmacokinetics of Continuous-Infusion Meropenem in Febrile Neutropenic Patients with Hematologic Malignancies: Dosing Strategies for Optimizing Empirical Treatment against Enterobacterales and *P. aeruginosa*

**DOI:** 10.3390/pharmaceutics12090785

**Published:** 2020-08-19

**Authors:** Pier Giorgio Cojutti, Anna Candoni, Davide Lazzarotto, Carla Filì, Maria Zannier, Renato Fanin, Federico Pea

**Affiliations:** 1 Department of Medicine, University of Udine, 33100 Udine, Italy; piergiorgio.cojutti@uniud.it (P.G.C.); renato.fanin@uniud.it (R.F.); 2 Institute of Clinical Pharmacology, Santa Maria della Misericordia University-Hospital of Udine, Azienda Sanitaria Universitaria Friuli Centrale (ASUFC), 33100 Udine, Italy; 3 Division of Haematology, Santa Maria della Misericordia University-Hospital of Udine, Azienda Sanitaria Universitaria Friuli Centrale (ASUFC), 33100 Udine, Italy; anna.candoni@asufc.sanita.fvg.it (A.C.); davide.lazzarotto@asufc.sanita.fvg.it (D.L.); carla.fili@asufc.sanita.fvg.it (C.F.); mariaelena.zannier@asufc.sanita.fvg.it (M.Z.)

**Keywords:** continuous-infusion meropenem, patients with hematologic malignancies, population pharmacokinetics

## Abstract

A population pharmacokinetic analysis of continuous infusion (CI) meropenem was conducted in a prospective cohort of febrile neutropenic (FN) patients with hematologic malignancies. A non-parametric approach with Pmetrics was used for pharmacokinetic analysis and covariate evaluation. Monte Carlo simulations were performed for identifying the most appropriate dosages for empirical treatment against common Enterobacterales and *P. aeruginosa.* The probability of target attainment (PTA) of steady-state meropenem concentration (Css)-to-minimum inhibitory concentration (MIC) ratio (Css/MIC) ≥1 and ≥4 at the European Committee on Antimicrobial Susceptibility Testing (EUCAST) clinical breakpoint of 2 mg/L were calculated. Cumulative fraction of response (CFR) against Enterobacterales and *P. aeruginosa* were assessed as well. PTAs and CFRs ≥ 90% were considered optimal. A total of 61 patients with 178 meropenem Css were included. Creatinine clearance (CL_CR_) was the only covariate associated with meropenem clearance. Monte Carlo simulations showed that dosages of meropenem ranging between 1 g q8h and 1.25 g q6h by CI may grant optimal PTAs of Css/MIC ≥4 at the EUCAST clinical breakpoint. Optimal CFRs may be granted with these dosages against the Enterobacterales at Css/MIC ≥ 4 and against *P. aeruginosa* at Css/MIC ≥ 1. When dealing against *P. aeruginosa* at Css/MIC ≥ 4, only a dosage of 1.5 g q6h by CI may grant quasi-optimal CFR (around 80–87%). In conclusion, our findings suggest that dosages of meropenem ranging between 1 g q8h and 1.25 g q6h by CI may maximize empirical treatment against Enterobacterales and *P. aeruginosa* among FN patients with hematologic malignancies having different degree of renal function.

## 1. Introduction

Patients with hematologic malignancies when suffering from febrile neutropenia (FN) may be at increased risk of developing bacterial infectious complications. Bloodstream infections (BSI) are among the most common and severe ones, with prevalence rates ranging between 11 and 38% [1]. During the last decade, Gram-negative bacteria have become the most prevalent etiological agents of BSI in patients with hematologic malignancies. Two large European epidemiologic studies showed that Gram-negatives accounted for 49–52.6% of BSIs and Gram-positives for 41–46.6% of cases [2,3]. *Escherichia coli*, *Klebsiella pneumoniae*, *Enterobacter cloacae* and *Pseudomonas aeruginosa* are the most common Gram-negative isolates [3]. Decreased antimicrobial susceptibility is increasingly reported among these pathogens and may represent a worrisome concern in the clinical management of these infections [4]. The overall susceptibility rates of Gram-negatives isolated from patients with hematologic malignancies were 69.8% to cephalosporins, 79.1% to piperacillin/tazobactam and 63.1% to meropenem [3].

Current guidelines recommend an antipseudomonal beta-lactam, such as piperacillin/tazobactam, cefepime or ceftazidime, as first-line choice for empirical treatment of FN patients with hematologic malignancies. Escalation to meropenem is suggested in the absence of a clinical response within 48–72 h [5,6].

Meropenem is a beta-lactam antibiotic whose effect is achieved by the duration of time the serum concentration of the antibiotic is above the minimum inhibitory concentration of the microorganism (time above MIC). This effect is maximally achieved when the concentration of meropenem exceeds the MIC for at least 40% of the dosing interval [7].

However, when dealing with severe infections in immunocompromised hosts and/or in critically ill patients, a more conservative pharmacodynamic target of efficacy up to 100% t > 4–6 × MIC is highly advocated both for maximizing efficacy [8,9] and for preventing the development of breakthrough resistance as well [10]. Administration by continuous infusion (CI) may be helpful in attaining higher pharmacodynamic targets with meropenem in the empirical treatment of FN patients [11]. 

The aim of this study was to conduct a population pharmacokinetic analysis of CI meropenem in FN patients with hematologic malignancies and to identify dosing strategies that may be helpful in maximizing efficacy and in preventing resistance development in the empirical treatment of Gram-negative infections with meropenem.

## 2. Materials and Methods 

### 2.1. Study Design

Data for this analysis came from a recent prospective, monocentric, interventional study that assessed the role of real-time therapeutic drug monitoring (TDM)-based optimization of CI meropenem in improving treatment outcome among FN patients with hematologic malignancies [12].

In brief, after starting treatment with fixed meropenem dosing regimen (1 g loading dose over 30 min followed by a maintenance dose of 1 g q8h CI over 8 h if creatinine clearance (CL_CR_) ≥ 60 mL/min/1.73 m^2^ or 0.5 g q6h CI over 6 h if CL_CR_ < 60 mL/min/1.73 m^2^), patients underwent real-time TDM finalized at achieving meropenem steady-state plasma concentrations (Css) of 8–16 mg/L [13] (namely a Css/MIC ratio of 4–8 fold the EUCAST clinical breakpoint of 2 mg/L against Enterobacterales and *P. aeruginosa*). TDM was first assessed on days 2–3 and then reassessed every 48–72 h until the end of treatment. Stability of CI meropenem was granted by reconstitution of the aqueous solution every 6–8 h with infusion over 6–8 h [14].

Peripheral blood samples were drawn at each TDM assessment, and meropenem concentrations were analyzed by means of a validated high-performance liquid chromatography (HPLC) method [15] with some modifications, as previously described [13,16]. Precision and accuracy were assessed by replicate analysis of quality control samples against calibration standards. Intra- and inter-assay coefficient of variation was always <10%. The lowest limit of detection was 0.5 mg/L.

Patient clinical data (age, gender, weight, height, type of hematologic disease, type and site of infection) were recorded at baseline. Serum creatinine was collected at each TDM assessment, and CL_CR_ was estimated by means of the Chronic Kidney Disease Epidemiology (CKD-EPI) formula [17]. Patient outcome was defined at end of treatment as cured (when all the following occurred: fever disappearance for >48 h, microbiologically eradication with negative cultures in at least two subsequent assessments (in case of documented infection), no radiological signs of infections, no change of antimicrobial therapy) or failed.

### 2.2. Population Pharmacokinetic Modelling

Population pharmacokinetic analysis was conducted using the non-parametric adaptive grid (NPAG) approach and the algebraic model solver included in the Pmetrics package(version 1.5.0; Laboratory of Applied Pharmacokinetics and Bioinformatics, Los Angeles, CA, USA) of R (version 3.4.4) [18]. A one-compartment base model with zero-order administration and first-order elimination from the central compartment was developed. Pharmacokinetic models with multiple compartments were not tested as we deemed that concentration-time data obtained during continuous-infusion administration did not allow for an accurate estimation of the volume of distribution. Maximum a posteriori (MAP) Bayesian estimates of meropenem clearance (CL) and volume of distribution (V) were determined in each patient.

Influence of covariates was assessed by including the biologically plausible clinical covariates (age, height, weight, gender, CL_CR_) into the basic model. The degree of association between each covariate and the median MAP Bayesian estimates of meropenem pharmacokinetic parameters was assessed by means of linear regression and of the forward/backward elimination. Variability in the continuous covariates included in the final model was considered by splitting each covariate distribution into homogenuous classes according to the frequency observed in the study population. Covariates were normally distributed within each class and centered around their mean ± SD.

Comparisons of the performances of the models were evaluated by calculating the objective function value (OFV), as well as the Akaike information criteria (AIC) and the Bayesian information criteria (BIC). A decrease of at least 3.84 points in the OFV coupled with a decrease of the AIC and the BIC values were considered for adding the covariates into the basic model.

The goodness of fit and the coefficient of determination of the linear regression of the observed versus the population predicted and individual predicted plot were considered for defining the final population pharmacokinetic model. Internal model validation was performed by means of a visual predictive check (VPC) and by calculating the normalized prediction distribution errors (NPDE). The VPC plot is based on 1000 simulations per each subject in the original population, and by overlaying the observed plasma concentrations with the 95% CIs of the simulated 5th, 25th, 50th, 75th and 95th percentiles. NPDE were calculated for providing a quantitative assessment of the final model. This tool was preferred compared with weighted residual plots because it is considered more reliable for evaluating pharmacokinetic model including covariates [19]. The distribution of NPDEs should be normal in the presence of appropriateness of model fit.

Assay error in the population model was estimated by means of the inter-day variability of laboratory assay data. A first-order polynomial relationship between drug concentrations and the standard deviation of the observations was used (C0 = 0.224, C1 = 0.060). Extra process noise was captured with a gamma (G) model (G = 5).

### 2.3. Monte Carlo Simulation Analysis and Probability of Target Attainment

One-thousand subject Monte Carlo simulations for each of six incremental dosing regimens of CI meropenem (0.25 g q6h CI, 0.5 g q6h CI, 1 g q8h CI, 1 g q6h CI, 1.25 g q6h CI and 1.5 g q6h CI) were conducted by means of Pmetrics. Meropenem Css were simulated at 48 h. The objective was that of assessing the probability of target attainment (PTA) of a Css/MIC ratio ≥1 and/or ≥4 at the EUCAST clinical breakpoint (2 mg/L) against the most common Enterobacterales and *Pseudomonas aeruginosa*. PTAs of ≥90% were considered as optimal for maximizing the efficacy of empirical treatment with meropenem in FN patients with hematologic malignancies.

The cumulative fraction of response (CFR) achievable with the tested CI meropenem dosages was calculated against the EUCAST MIC distributions for *E.coli*, *K. pneumoniae*, *E. cloacae* and *P. aeruginosa* [20]. CFRs ≥ 90% were considered as optimal.

### 2.4. Ethics

This study was approved by the Ethics Review Board of the Friuli-Venezia-Giulia Region (protocol number: 20496/CEUR, approved: 28 July 2017). Written informed consent was obtained from each patient before enrollment.

## 3. Results

### 3.1. Patient Population and Meropenem Therapy 

From a total of 100 patients who were enrolled in the prospective clinical study [12], 39 were excluded from this analysis because of the inadequacy of blood sampling. The demographic and clinical characteristics of the 61 definitive patients are summarized in Table 1. Median patient age, weight and CL_CR_ were 55 (IQR 54–60) years, 77 (IQR 63–85) kg and 107.3 (IQR 96.1–123.6) mL/min/1.73 m^2^, respectively. Acute myeloid leukemia was the most frequent underlying hematological disease (57.4%). Thirty out of 61 patients (49.2%) had clinically documented infections, pneumonia (36.7%) and BSI (33.3%) accounting for most of them. Only seven patients had documented Gram-negative infections. Median CI meropenem dose was of 1 g q8h CI. The median duration of meropenem therapy was nine days (IQR 7–12.3 days) and the median number of TDM assessments per patient was 3 (IQR 3–4). The majority of patients (91.8%) were cured.

### 3.2. Population Pharmacokinetic Analysis 

A total of 178 plasma meropenem Css were included in the population pharmacokinetic model (median (IQR) value of 10.5 (8.3–10.2) mg/L). The one-compartment base model provided a high fit to the data (R^2^ of the observed versus predicted concentrations of 0.786), with OFV, BIC and AIC of 928.9, 935 and 944, respectively. 

The only covariate significantly associated with meropenem CL was CL_CR_. After inclusion of CL_CR_ into the base model, the R^2^ regression value of the observed vs. individual predicted concentrations increased to 0.849, and the values of OFV, BIC and AIC decreased to 874.4, 852 and 895, respectively. 

**Figure 1:**
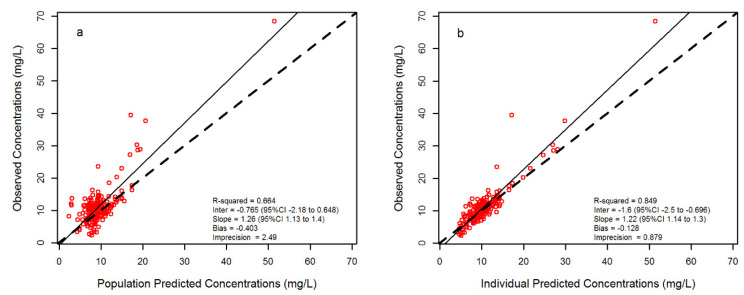
Scatter and linear fit plot for the final population pharmacokinetic model. Observed versus population-predicted plasma concentrations (**a**) and observed versus individual-predicted plasma concentrations (**b**). Solid lines are the lines of regression between observed and predicted concentrations. Dashed lines are the lines of identity. Red rings are meropenem concentrations.

As shown in Figure 1, the individual model-based predictions may be considered appropriate since it deviated from unity essentially in presence of very high concentrations (approximately > 20 mg/L), which represented only a minority of the overall measurements (5.62%). Bias and imprecision were acceptable (−0.128 and 0.879, respectively).

The final model was as follows:

CLi = θ_1_ + θ_2_ × CL_CRi_ (1)

where CLi is meropenem clearance of the ith subject, θ_1_ is the clearance (intercept) when CL_CR_ = 0, θ_2_ is the slope estimate reflecting the change in clearance per unit change in CL_CR_, and CL_CRi_ is the creatinine clearance of the ith subject.

**Figure 2:**
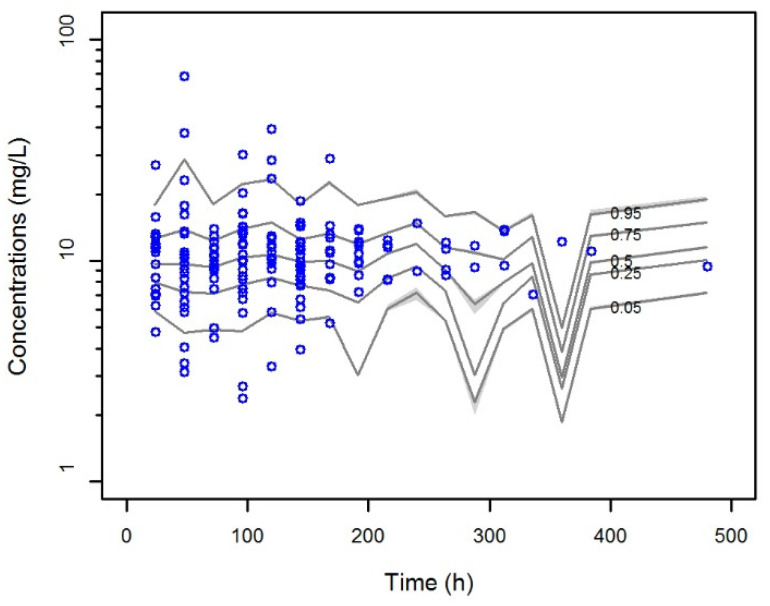
Visual predictive check (VPC) of meropenem concentration versus time for the final covariate model. Gray shadings display predicted interval of simulated data. Blue dots represent observed concentrations.

The VPC of the final model (Figure 2) showed that 87.6% of the observed concentrations reside within the 95% confidence intervals derived from model predictions. The weighted residuals were normally distributed around zero (p = 0.219 with the Shapiro-Wilk test).

Table 2 summarizes the statistics of the population Bayesian pharmacokinetic parameters obtained with the final covariate model. The mean (±SD) population pharmacokinetic estimates of the final multivariate model were CL = 13.04 (4.85) L/h and V = 21.88 (5.85) L.

### 3.3. Monte Carlo Simulation and the Probability of Target Attainment

**Figure 3:**
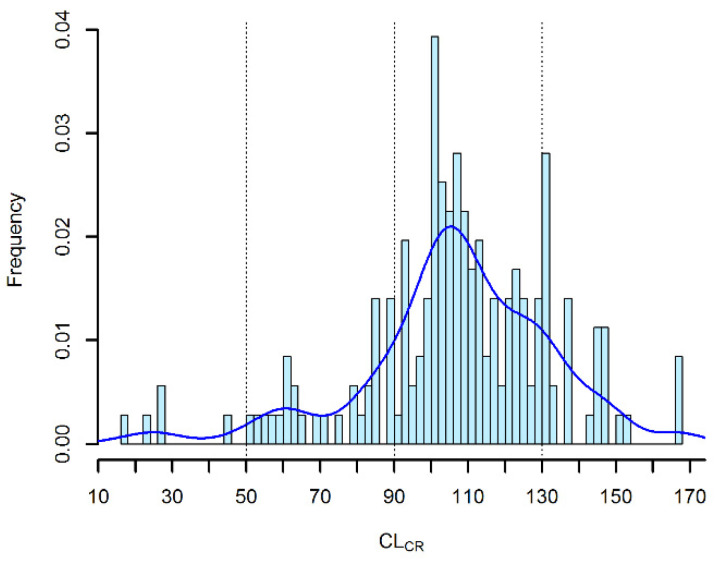
Histogram and kernel density plot of the distribution of patients’ creatinine clearance (CL_CR_).

The frequency of distribution of CL_CR_ observed in our patient population is depicted in Figure 3. Accordingly, 3 different classes of renal function were identified (decreased CL_CR_ (50–89 mL/min/1.73 m^2^); normal CL_CR_ (90–129 mL/min/1.73 m^2^); augmented renal clearance (ARC) (CL_CR_ ≥ 130 mL/min/1.73 m^2^)). On this basis, a total of 18 one-thousand Monte Carlo simultions were conducted in order to test six incremental dosing regimens of CI meropenem (0.25 g q6h CI, 0.5 g q6h CI, 1 g q8h CI, 1 g q6h CI, 1.25 g q6h CI and 1.5 g q6h CI) across these classes of renal function.

**Figure 4:**
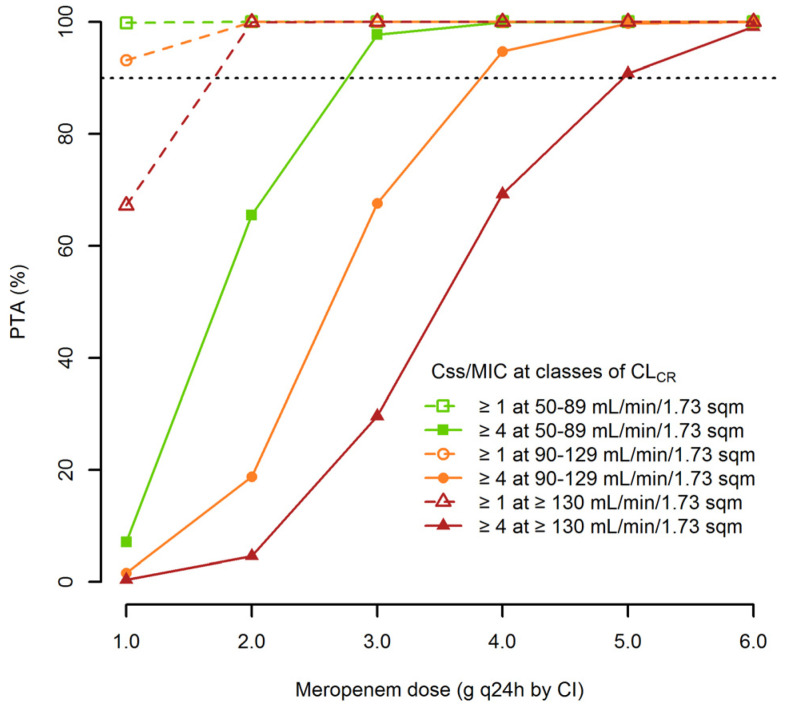
Probability of target attainments (PTAs) of Css/MIC ≥ 4 (solid lines) and Css/MIC ≥ 1 (dashed lines) at the EUCAST clinical breakpoint of 2 mg/L against Enterobacterales and *P. aeruginosa* with incremental dosages of continuous infusion meropenem in relation to different classes of CL_CR_. Horizontal broken line identifies the threshold for optimal PTA (≥90%).

Figure 4 shows the PTAs of Css/MIC ≥ 1 and Css/MIC ≥ 4 at the EUCAST clinical breakpoint for Enterobacterales and *P. aeruginosa* (2 mg/L) achievable with increasing dosages of CI meropenem among the different classes of CL_CR_. Optimal PTAs of Css/MIC ≥4 were granted by meropenem dosages of 1 g q8h CI, 1 g q6h CI and 1.25 g q6h CI in patients with CL_CR_ of 50–89, 90–129 and ≥130 mL/min/1.73 m^2^, respectively. Lower dosages (0.25 g q6h CI in patients with CL_CR_ of 50–89 and 90–129 mL/min/1.73 m^2^, 0.5 g q6h CI in those with ≥130 mL/min/1.73 m^2^) were sufficient for achieving optimal PTAs of Css/MIC ≥ 1.

The CFRs achievable at Css/MIC of ≥4 and ≥1 against the EUCAST MIC distributions of *E. coli*, *K. pneumoniae*, *E. cloacae* and *P. aeruginosa* with incremental dosages of CI meropenem in different classes of renal function are summarized in Table 3. Optimal CFRs against *E. coli*, *K. pneumoniae* and *E. cloacae* were granted at Css/MIC ≥4 just with dosing regimen as low as 0.25 g q6h CI in all of the classes of renal function. Optimal CFRs against *P. aeruginosa* were granted at Css/MIC ≥ 1 with meropenem dosages of 0.5 g q6h CI, 1 g q8h CI and 1 g q6h CI in patients with CL_CR_ of 50–89, 90–129 and ≥130 mL/min/1.73 m^2^, respectively. Conversely, when targeting at Css/MIC ≥ 4 against *P. aeruginosa*, only quasi-optimal CFRs, ranging between 87.78 and 81.30% were achievable with the highest dosing regimen of 1.5 g q6h CI across the three classes of CL_CR_. 

## 4. Discussion

In this study, we conducted a population pharmacokinetic analysis with CI meropenem in FN patients with hematologic malignancies and tested which could be the dosages that are most advisable for empirical treatment against *P. aeruginosa*.

To the best of our knowledge, this is the first study that assessed the population pharmacokinetics of meropenem in FN patients with hematologic malignancies during administration by CI at different dosages of the drug. The population pharmacokinetics of meropenem was investigated among FN patients with hematologic malignancies during intermittent infusion administration in two previous studies. Overall, our findings are in agreement with their results. Among 57 Korean patients who were treated with a meropenem dose of 0.5 g every 8 h by intermittent infusion and had a mean CL_CR_ of 121 mL/min, the estimates of CL and Vd were 9.7 L/h and 14.6 L, respectively [21]. Ariano et al. found that among 60 bacteremic patients with FN who received a meropenem dose of 1 g every 8 h by intermittent infusion and had a CL_CR_ ranging 97–107 mL/min/1.73 m^2^, the estimated CL was 15.4 L/h and the Vd was 14.4 L [11]. 

The association of CL_CR_ with meropenem CL is consistent with meropenem being eliminated mainly by the renal route and is in agreement with previous findings as well. 

From the pathophysiological standpoint, FN patients with hematologic malignancies may be considered as a special population. Previous studies showed that some underlying conditions may significantly alter the pharmacokinetic behavior of hydrophilic antibiotics in this population. The renal clearance of the aminoglycosides [22,23], the beta-lactams [24,25] and of daptomycin [26] were shown to be greatly increased in patients with acute leukemia. Noteworthy, as much as 22.9% of our study population had ARC at presentation, namely a condition that may increase meropenem CL. This may cause drug underexposure when standard dosages of meropenem are administered by intermittent infusion. 

Extended or continuous infusion administration was shown to be beneficial in attaining the pharmacodynamic target of efficacy with beta-lactams and in improving clinical outcome in the treatment of patients with FN [12,27] and of critically ill patients with severe infections as well [8,28,29]. Additionally, it is worth mentioning that in a recent prospective study focused at targeting meropenem Css/MIC at 4–8 by means of real-time TDM, we showed that this strategy may be helpful in FN patients with hematologic malignancies also in preventing the emergence of carbapenem resistance among Enterobacterales. No colonization by carbapenem-resistant Enterobacterales was found at rectal swabs among all of the patients (63) who were re-hospitalized within 3-months after meropenem treatment [12].

The need for high-dose regimens of meropenem by CI has been previously advocated for the treatment of severe infections in various settings [30,31,32]. Monte Carlo simulations showed that dosages of meropenem ranging between 1 g q8h and 1.25 g q6h by CI may maximize empirical treatment against Enterobacterales and *P. aeruginosa* among FN patients with hematologic malignancies and different degree of renal function. These dosages are reliable for achieving optimal PTAs of Css/MIC ≥ 4 against all of the bacterial strains that are susceptible to meropenem according to the EUCAST clinical breakpoint (≤2 mg/L). Additionally, they may grant optimal CFRs against the Enterobacterales at Css/MIC ≥ 4 and against *P.aeruginosa* at Css/MIC ≥ 1. When dealing against *P. aeruginosa* at Css/MIC ≥ 4, these dosages may be suboptimal, and only a dosage of 1.5 g q6h by CI may grant quasi-optimal CFR (around 80–87%) in all of the three classes of renal function. This is due to the fact that around 20% of the *P. aeruginosa* strains may have MIC > 2 mg/L according to the EUCAST MIC distribution [20] and are therefore considered carbapenem-resistant in vitro. When dealing with meropenem-resistant *P. aeruginosa* isolates, real-time TDM may allow optimization of meropenem treatment [12]. Alternatively, switch to other anti-pseudomonal agents, like ceftolozane-tazobactam, should be considered. 

We acknowledge some limits of this study. The limited number of TDM assessments per patient and estimated, rather than measured, renal function might account for some unexplained variability in the population pharmacokinetic model. However, the prospective design and the large sample size are valuable strengths of this study.

In conclusion, our findings suggest that dosages of meropenem ranging between 1 g q8h and 1.25 g q6h by CI may maximize empirical treatment against Enterobacterales and *P. aeruginosa* among FN patients with hematologic malignancies and different degree of renal function. Real-time TDM may represent a valuable tool for appropriately targeting meropenem Css in patients with ARC and/or with borderline susceptible *P. aeruginosa* strains. 

## Figures and Tables

**Table 1:** Patient characteristics.

Patient demographics		
	Total number of patients	61
	Age (years)	55 (54–60)
	Gender (male/female)	37/24
	Body weight (kg)	77 (63–85)
	CL_CR_ (mL/min/1.73 m^2^)	107.3 (96.1–123.6)
	Patients with ARC	14 (22.9)
Underlying hematological disease		
	AML	35 (57.4)
	Lymphoma	12 (19.7)
	ALL	11 (18.0)
	MM	3 (4.9)
Clinically documented infections		
	Overall	30 (49.2)
	Pneumonia	11 (18.0)
	BSI	10 (16.4)
	Intra-abdominal infection	5 (8.2)
	SSTI	2 (3.4)
	UTI	1 (1.6)
	Septic shock	1 (1.6)
Gram-negative isolates		
	*Escherichia coli*	5 (8.2)
	*Klebsiella pneumonia*	1 (1.6)
	*Pseudomonas aeruginosa*	1 (1.6)
Meropenem treatment		
	Median dose (g)	1 g q8h CI (1 g q8h CI–1 g q8h CI)
	Length of treatment (days)	9 (7–12.3)
	No. of TDM assessments per patient	3 (3–4)
	Meropenem Css (mg/L)	10.5 (8.3–10.2)
Clinical outcome		
	Cured	56 (91.8)
	Failed	5 (8.2)

Data are presented as median (IQR) for continuous variables, and as number (%) for dichotomous variables. ALL, acute lymphocytic leukemia; AML, acute myeloid leukemia; ARC, augmented renal clearance (defined as CL_CR_ ≥ 130 mL/min/1.73 m^2^); BSI, blood stream infection; CL_CR_, creatinine clearance; MM, multiple myeloma; SSTI, skin and soft tissue infection; TDM, therapeutic drug monitoring; UTI, urinary tract infection.

**Table 2:** Parameter estimates of meropenem for the final covariate one-compartment population pharmacokinetic model.

Parameter	Mean	Standard Deviation	Coefficient of Variation (%)	Median
CLi (L/h) = θ1 + θ2 × CLCRi				
θ1	0.27	0.13	48.53	0.20
θ2	0.12	0.03	27.44	0.13
V (L)	21.88	5.85	26.71	20.00

θ_1_ and θ_2_ are the intercept and slope, respectively, of the linear relationship between meropenem clearance of the ith subject (CLi) and creatinine clearance of the ith subject (CL_CRi_) estimated by means of the CKD-EPI formula; V, volume of distribution.

**Table 3:** Cumulative fraction of response (CFR) achievable at Css/MIC of ≥4 and ≥1 against the EUCAST MIC distributions of *E. coli*, *K. pneumoniae*, *E. cloacae* and *P. aeruginosa* with incremental dosages of CI meropenem in different classes of renal function.

CI-Meropenem Dosages at Classes of Renal Function	*E. coli*		*K. pneumoniae*		*E. cloacae*		*P. aeruginosa*	
	Css/MIC ≥ 4	Css/MIC ≥ 1	Css/MIC ≥ 4	Css/MIC ≥ 1	Css/MIC ≥ 4	Css/MIC ≥ 1	Css/MIC ≥ 4	Css/MIC ≥ 1
CL_CR_: 50–89 mL/min/1.73 m^2^								
0.25 g q6h CI	99.92	99.97	99.04	99.64	98.79	99.58	65.83	84.14
0.5 g q6h CI	99.96	99.98	99.40	99.80	99.33	99.75	76.71	90.11
1 g q8h CI	99.97	99.97	99.55	99.88	99.50	99.86	81.41	93.72
1 g q6h CI	99.97	100.00	99.64	99.93	99.57	99.92	84.08	96.15
1.25 g q6h CI	99.98	100.00	99.70	99.96	99.63	99.95	86.13	97.74
1.5 g q6h CI	99.9	100.00	99.75	99.98	99.68	99.97	87.78	98.57
CL_CR_: 90–129 mL/min/1.73 m^2^								
0.25 g q6h CI	99.86	99.96	98.72	99.52	98.24	99.46	56.76	80.12
0.5 g q6h CI	99.95	99.98	99.23	99.72	99.09	99.65	71.25	86.71
1 g q8h CI	99.96	99.98	99.41	99.81	99.34	99.76	76.91	90.21
1 g q6h CI	99.96	99.99	99.52	99.86	99.46	99.83	80.20	92.59
1.25 g q6h CI	99.97	99.99	99.58	99.90	99.53	99.88	82.28	94.47
1.5 g q6h CI	99.97	100.00	99.64	99.93	99.57	99.92	84.21	96.30
CL_CR_: ≥130 mL/min/1.73 m^2^								
0.25 g q6h CI	99.80	99.96	98.43	99.41	97.62	99.33	48.90	76.66
0.5 g q6h CI	99.92	99.97	99.05	99.64	98.81	99.57	66.20	84.17
1 g q8h CI	99.95	99.99	99.28	99.74	99.17	99.67	77.91	87.62
1 g q6h CI	99.96	99.99	99.41	99.81	99.34	99.75	76.87	90.14
1.25 g q6h CI	99.96	99.99	99.49	99.85	99.44	99.81	79.60	92.13
1.5 g q6h CI	99.97	99.99	99.55	99.88	99.49	99.85	81.30	93.59

CL_CR_, creatinine clearance; CI, continuous infusion.
